# Interaction of pAsa5 and pAsa8 Plasmids in *Aeromonas salmonicida* subsp. *salmonicida*

**DOI:** 10.3390/microorganisms11112685

**Published:** 2023-11-02

**Authors:** Pierre-Étienne Marcoux, Sarah B. Girard, Kim C. Fournier, Catherine A. Tardif, Ariane Gosselin, Steve J. Charette

**Affiliations:** 1 Institut de Biologie Intégrative et des Systèmes (IBIS), Université Laval, Quebec City, QC G1V 0A6, Canada; pierre-etienne.marcoux.1@ulaval.ca (P.-É.M.); kim.fournier.3@ulaval.ca (K.C.F.);; 2 Département de Biochimie, de Microbiologie et de Bio-Informatique, Faculté des Sciences et de Génie, Université Laval, Quebec City, QC G1V 0A6, Canada; 3 Centre de Recherche de L’Institut Universitaire de Cardiologie et de Pneumologie de Québec (IUCPQ), Quebec City, QC G1V 4G5, Canada

**Keywords:** pAsa5, pAsa8, *Aeromonas salmonicida* subsp. *salmonicida*, plasmid, recombination, transposon, Tn*1721*

## Abstract

The plasmid known as pAsa5 is present in *Aeromonas salmonicida* subsp. *salmonicida*, a fish pathogen. The pAsa5 plasmid carries genes that are essential for the bacterium’s virulence. Recombination events are known to occur in pAsa5, resulting in the loss of certain segments or the acquisition of additional genetic elements. For example, the transposon carried by the large pAsa8 plasmid was found to be inserted into the pAsa5 plasmid in the SHY16-3432 strain, enabling the addition of antibiotic resistance genes to this plasmid, which does not normally possess any. In this study, we present the isolation of additional strains carrying pAsa8. Further analyses of these strains revealed that a fusion between pAsa5 and the complete version of pAsa8 is possible. The pAsa8 transposon insertion in pAsa5 seen in the SHY16-3432 strain appears to be an aberrant event compared to the fusion of the two full-length plasmids. A 22-nucleotide sequence, present in both plasmids, serves as the site for the fusion of the two plasmids. Moreover, it is possible to introduce pAsa8 through conjugation into naive strains of *A. salmonicida* subsp. *salmonicida* and once the plasmid is within a new strain, the fusion with pAsa5 is detectable. This study reveals a previously unexplored aspect of pAsa5 plasmid biology, highlighting an additional risk for the spread of antibiotic resistance genes in *A. salmonicida* subsp. *salmonicida.*

## 1. Introduction

*Aeromonas salmonicida* subsp. *salmonicida*, a Gram-negative bacterium, exhibits psychrophilic characteristics, thriving optimally at around 18 °C. This subspecies is notably responsible for furunculosis, a disease that predominantly affects salmonid fish [1].

It is well known that the genome of *A. salmonicida* subsp. *salmonicida* includes various plasmids. Usually, the strains of this subspecies carry three small cryptic plasmids (pAsa1, pAsa2, and pAsa3) and a large plasmid named pAsa5 [2]. Additionally, a significant proportion of strains carry the small pAsal1 plasmid, which contributes to the virulence of the bacteria [3,4].

pAsa5 is a plasmid of 155 kbp that contains a segment of genes encoding the Type Three Secretion System (TTSS) [5], which is a needle-like protein structure that allows for the translocation of toxins from the cytoplasm of the bacteria to the cytoplasm of the host cells [6]. This system is an essential virulence factor for *A. salmonicida* subsp. *salmonicida* to be able to infect fish [7].

Numerous plasmids of varying sizes can be found in different strains of *A. salmonicida* subsp. *salmonicida*, and several of them contain antibiotic resistance genes (ARGs), which are often found in transposons and other mobile genetic elements [2]. One such plasmids is pAsa8 (1106 kbp), which contains genes that are likely involved in conjugative transfers. This plasmid confers resistance to a wide range of antibiotics, including tetracyclines, florfenicol, chloramphenicol, sulfonamides, ampicillin, carbenicillin, streptomycin, spectinomycin, and quaternary ammonium compounds. All of the ARGs that are present in pAsa8 are located on a mobile element derived from the Tn*1721* transposon, which includes a Class 1 integron [8,9].

Various examples of plasmid recombination events in *A. salmonicida* subsp. *salmonicida* have been described. One study demonstrated the excision of a transposon from its host plasmid, pAsa-2939, affecting the host bacterium’s antibiotic resistance. Interestingly, this excision of the transposon was observed when the plasmid host was *A. salmonicida* subsp. *salmonicida*, but not when it was *Aeromonas hydrophila* [10]. The TTSS locus on pAsa5 is also known to excise from the plasmid. In this case, the loss occurs when the bacterium is grown at temperatures of 25 °C and above due to recombination events involving the insertion sequences flanking the TTSS locus [11,12]. These pAsa5 rearrangements involve two types of insertion sequences (IS*As11* and IS*As5*), both resulting in various versions of the recombined plasmid, all lacking the TTSS locus [13]. Another case of recombination has been observed between pAsa5 and pAsa9 through the recombination between the IS*As5* copies found on both plasmids, leading to the fusion of the two plasmids [13]. Various mobile DNA elements can also be inserted into pAsa5. For example, various versions of pAsa5 with or without an IS*As5* near the conjugative genes are known, and when the IS*As5* is found in this region of the plasmid, at least two different insertion sites are known so far [8,13,14].

The most intriguing insertion observed so far in pAsa5 is that of the Tn*1721* transposon from pAsa8. This was observed in a single strain (SHY16-3432) isolated in Quebec, Canada. The resulting plasmid variant, named pAsa5-3432, retains all pAsa5 genes, except for three that were likely lost during the insertion process [8].

Given the frequent involvement of plasmids and mobile DNA elements in the transfer of ARGs, the objective of this study, by taking advantage of the discovery of additional strains carrying pAsa8, was to determine if the Tn*1721* located on pAsa8 can transpose into pAsa5, as found with the pAsa5-3432 variant. Based on the PCR results, we discovered the presence of only one of the two junctions between the transposon and the pAsa5. After further analyses with Sanger sequencing, PCR genotyping, and conjugation experiments, it appeared that the fusion of the full-length pAsa8 with pAsa5 was not only observed but may even be the most predominant form of recombination between these two plasmids, rather than the version involving only the transposon, as in the case of pAsa5-3432. The successful introduction of pAsa8 into strains lacking this plasmid demonstrated that recombination between pAsa5 and pAsa8 is possible as soon as these two plasmids coexist in the same cell.

## 2. Materials and Methods

### 2.1. Bacterial Strains

The *A. salmonicida* subsp. *salmonicida* strains used in this study are described in Table 1. They were cultivated from frozen stocks on furunculosis agar (FA) for a period of 3 days at 18 °C, in accordance with established procedures, as previously described [15].

### 2.2. PCR Analyses

The DNA lysates of the different strains were obtained by following a previously described protocol where bacterial cells are lysed via the action of detergents and heat [18]. The PCR mixture contained 4 μL of 5X Go-Taq buffer (Promega, Madison, WI, USA), 1.6 μL of 2 mM dNTP, 1.3 μL of each primer (100 ng/μL of each), 0.1 μL of GoTaq 5 U (Promega, Madison, WI, USA), 10.7 μL of H_2_O, and 1 μL of DNA template. The primers are shown in Table S1, and the PCR conditions used in this study are shown in Table S2. The DNA amplification products were separated via electrophoresis on 1% agarose gels, which were stained with 0.5 μg/mL ethidium bromide and viewed and photographed under a UV lamp. The PCR reactions were performed at least twice with the appropriate controls.

### 2.3. DNA Extraction and Sequencing

The total genomic DNA of the SHY20-5455 and SHY18-3658 strains were extracted using DNeasy Blood and Tissue kits (Qiagen, Montreal, QC, Canada) with the addition of an RNase A treatment step (20 µg/mL, Ambion, ThermoFisher, Saint-Laurent, QC, Canada) according to the manufacturer’s protocol. Sequencing libraries were prepared from purified bacterial DNA using the Nextera XT DNA Library Preparation Kit. The sequencing was performed using a MiSeq instrument (Illumina, San Diego, CA, USA) system at the Plateforme d’Analyse Génomique of the Institut de Biologie Intégrative et des Systèmes (Université Laval, Quebec City, QC, Canada).

### 2.4. Sequence Analysis

The sequencing reads of SHY18-3658 and SHY20-5455 were de novo assembled using A5-miseq version 20,160,825 [19]. Then, CONTIGuator version 2.7.5 was used to map the contigs on the reference genome of the A449 strain (NC_009348.1) [20]. The pAsa8 sequence obtained was then inserted into pAsa5 at a position equivalent to the insertion site of the pAsa8 transposon in pAsa5-3432 (NZ_CP038103.1). The coverage of the junction, the plasmid, and the chromosome using the Illumina reads was carried out using the bwa program (0.7.17) [21]. The reads were mapped against seven sequences; three of them (*dnaA* (WP_011898201), *P5G011* (WP_005321623), *tetA* (WP_000804064)) were from the NCBI database. The other four sequences were from an in silico model of pAsa5, which includes the complete sequence of pAsa8. Then, Qualimap (v2.2.2) was used for the statistical analyses [22].

### 2.5. Bacterial Conjugation Assays

The bacterial conjugation assays were performed as previously described [23]. The goal was to introduce pAsa8 into *A. salmonicida* subsp. *salmonicida* strains that did not have it. We could not use counter-selection with an antibiotic due to the large number of ARGs carried by pAsa8. Therefore, to recover only the recipient strains after conjugation, we used recipient strains that have no antibiotic resistance genes and are able to grow at a higher temperature (32 °C) than the maximum growth temperature of the donor strain (28 °C). These thermoadapted (TA) strains were produced by exposing parental JF2506 and JF2507 strains (Table 1) to 12 cycles of growth condition in tryptic soy broth where the temperature varied, as shown in Figure S1. The JF2506-TA and JF2507-TA strains produced in this way can grow at 32 °C, whereas this is not the case for the donor SHY20-5455 strain. In addition, considering the possible instability of the TTSS region on pAsa5 when the bacteria are cultured at higher temperatures than 25 °C, the presence of different genes on pAsa5 was verified in the JF2506-TA and JF2507-TA strains via a multiplex PCR described in the past [24]. PCR analyses were performed to detect the presence of pAsa8 in transconjugants using the primers listed in Table S1. The absence of the donor strain was confirmed via PCR with primers that target a genomic island named *AsaGEI1a*, which is not present in the recipient strains, but is present in the donor strain [25].

## 3. Results

**Figure 1:**
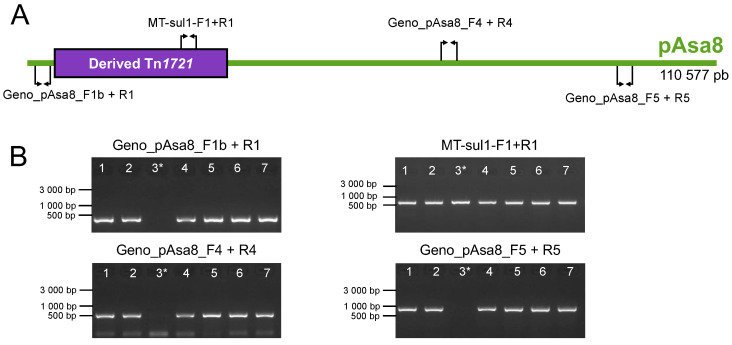
Genotyping of pAsa8 in *A. salmonicida* subsp. *salmonicida* strains from Québec. (**A**) Schematic and linear representation of pAsa8 based on the reference sequence (GenBank: KX364409). The purple section represents the derived Tn*1721* transposon, and the small arrows correspond to the location of the primers used for genotyping. (**B**) PCR results for the 4 pairs of primers used to detect the backbone of the pAs8 and the transposon. The strains tested are in the following order (lanes 1 to 7): M15448-11, M16474-11, SHY16-3432, SHY18-3658, SHY20-5455, SHY21-2359, and SHY21-3158. The SHY16-3432 (3*) strain does not have pAsa8, but only its transposon in pAsa5.

Following the characterization of new strains of *A. salmonicida* subsp. *salmonicida* from Quebec (Canada) in the past 5 years, we have now identified four additional strains that bear pAsa8 (SHY18-3658, SHY20-5455, SHY21-2359, and SHY21-3158). In total, there are now six strains of *A. salmonicida* subsp. *salmonicida* with pAsa8 in our repertoire, including the two strains described in the study by Trudel et al., 2016 (M15448-11 and M16474-11) and the four new ones presented in this study (Table 1). The presence of pAsa8 was confirmed in these strains via PCR genotyping using the primers listed in Table S1. The PCR results show that the complete structure of pAsa8 is present in the four new strains. Indeed, all four primer pairs targeting different locations on the plasmid generated the predicted amplifications (Figure 1). As expected, the SHY16-3432 strain is negative for pAsa8 genes since it contains only the Tn*1721* transposon but not the backbone of the plasmid.

The genomes of the SHY20-5455 and SHY18-3658 strains were sequenced using the Illumina method to have additional data sets for subsequent analyses on top of the sequencing data already available for the M16474-11 strain, which also bears pAsa8 [9]. The availability of these new strains that carry pAsa8, along with the sequencing data, opens the door to further analyses of the biology of this plasmid.

**Figure 2:**
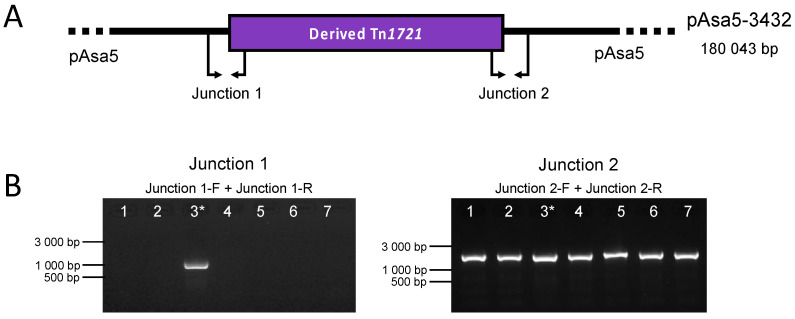
Genotyping of strains that bear a pAsa8 and a pAsa5 for the transposition of the derived Tn*1721*. (**A**) Schematic representation of the pAsa5-3432 variant found in SHY16-3432 strain showing the derived Tn*1721* (in purple) with both junctions flanking the transposon. The smaller arrows at the top of the figure named Jct 1-F, Jct 1-R, Jct 2-F, and Jct 2-R represent the primers used to detect the presence of the junction. (**B**) The PCR results from the 6 strains bearing pAsa8 and pAsa5 and the SHY16-3436 (3*) strain. The strains tested are in the following order (lanes 1 to 7): M15448-11, M16474-11, SHY16-3432, SHY18-3658, SHY20-5455, SHY21-2359, and SHY21-3158.

Massicotte et al. (2019) observed that the Tn*1721* transposon of pAsa8 is completely inserted into the pAsa5 of the SHY16-3432 strain, creating the pAsa5-3432 variant. In the current study, we wanted to determine whether the transposition of Tn*1721* into pAsa5 would be possible when the strain contains a full-length version of pAsa8. We performed PCR using primers that detect the insertion of the Tn*1721* transposon of pAsa8 in pAsa5. Each of the primer pairs used target one of the transposon insertion sites (Junction 1 and Junction 2) (Table S1) [8]. For this analysis, the SHY16-3432 strain that bears pAsa5-3432 was used as a control. The PCR results showed that all strains containing the full-length pAsa8 were positive for Junction 2 and negative for Junction 1 (Figure 2). Only the SHY16-3432 strain gave a positive signal for both junctions. Since only one of the two junctions gave a positive signal, we deduced that the Tn*1721* transposon is inserted in a different way than expected.

**Figure 3:**
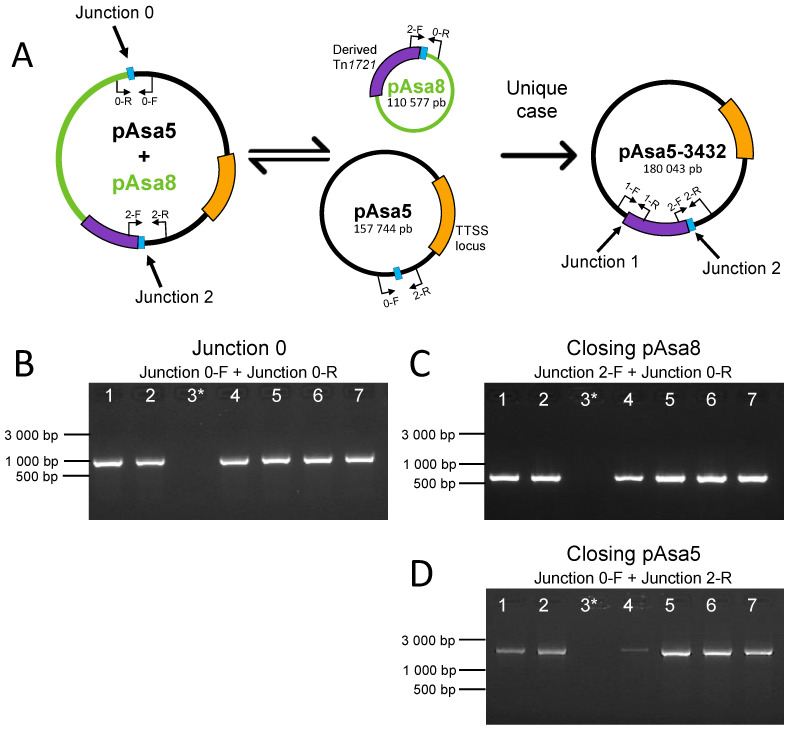
Insertion of pAsa8 inside pAsa5. (**A**) Schematic representation of the fusion between pAsa8 and pAsa5. The smaller arrows represent the primers, and the blue rectangle represents the location of the 22-nucleotide sequence. (**B**) PCR results for Junction 0, obtained using the primer Junction 0-F and Junction 0-R. The SHY16-3432 strain is used as a negative control (line 3*). The 6 other strains are the strains that bear pAsa8 and pAsa5. The tested strains are arranged as follows (lane 1 to 7): M15448-11, M16474-11, SHY16-3432, SHY18-3658, SHY20-5455, SHY21-2359, and SHY21-3158. (**C**) PCR results for self-closure of pAsa8 using Junction 2-F and Junction 0-R primers, with strains following the same order as in (**B**). (**D**) PCR results for self-closure of pAsa5 using Junction 0-F and Junction 2-R primers, with strains following the same order as in (**B**).

We therefore hypothesized that the full-length versions of the two plasmids may be fused together, like what was previously observed for the fusion of pAsa5 with pAsa9 [13]. Using bioinformatics tools, we created an in silico variant of pAsa5 that included the complete sequence of pAsa8 based on the result of the PCR that targeted Junction 2. We then created primers on both pAsa5 and pAsa8 sequences (Table S1) to verify the presence of this new junction (named Junction 0). All of the strains tested except for SHY16-3432 gave positive signals for this new primer pair (Figure 3), which backs up the hypothesis of the fusion of full-length pAsa8 with pAsa5.

Following the positive results for Junction 2 and Junction 0, we performed Sanger sequencing for the corresponding amplicons for each strain that contained pAsa8. For both junctions, we compared the sequence obtained from Sanger to the in silico variant of pAsa5. The identity percentage for Junction 0 was 97%, and for Junction 2, it was 100%. The sequence analysis revealed that the 3% missing was in fact a gap of a 22-nucleotide sequence, 5′-TTTGGCCGTTGGCCTAGACTCC-3′, which was present at each junction in the fused plasmid and not included in our in silico model of the fused plasmid. We discovered that this same 22-nucleotide sequence is present in both plasmids (pAsa5 and pAsa8) in the regions involved in the recombination between the two plasmids (Figure 3A). 

When we compared the pAsa5-3432 sequence with a reference sequence of pAsa5 (A449), we noticed a region of around 5 kpb that was deleted compared to the reference sequence [8]. This region contains three genes, two of which code for hypothetical proteins and one that codes for the KrfA protein involved in the regulation of plasmid segregation [8,26]. Using PCR, we confirmed that strains with a full-length pAsa8 (M15448-11, M16474-11, SHY16-3432, SHY18-3658, SHY20-5455, SHY21-2359, and SHY21-3158) have the three pAsa5 genes. In other words, while the insertion of the pAsa8-Tn*1721* transposon into pAsa5 in the SHY16-3432 strain results in gene loss in pAsa5, this does not occur when we observe a fusion of the full-length versions of pAsa5 and pAsa8.

Following the confirmation of the presence of the fusion between the two plasmids, we wanted to know whether the plasmids could also be present as independent replicons in the strain. The primers used to detect Junction 0 and Junction 2 (Figure 3A) were used in different combinations to detect the unfused forms of the two plasmids (Table S2). For pAsa5, the PCR results showed that pAsa5 was also present without the pAsa8 insertion, and that strains also contain pAsa8 independently (Figure 3C,D). Figure 3D shows a weaker but visible PCR signal for the SHY18-3658 strain compared to other strains with full-length pAsa8 and pAsa5. 

Knowing that the PCR results showed that the strains contained both a fused and a non-fused form, we wanted to find out if there was a predominant form. To determine the proportion of each version, we used the coverage of Illumina reads for the M16474-11, SHY18-3658, and SHY20-5455 strains. The sequencing data set from the SHY15-2939 strain was used as a negative control because it does not contain pAsa8 [10]. For the fusion between pAsa5 and pAsa8, we mapped the reads against Junction 2 and Junction 0 to compare against the sequence of both plasmids without the insertion. We also compared a gene on pAsa5 (*P5G011*) and a gene on pAsa8 (*tetA*) against a chromosomal gene (*dnaA*). As shown in Table 2, the coverage of Junction 2 and Junction 0 seem to be a little lower than the closing sequence of pAsa5 and pAsa8. However, the standard deviation for the two junctions and the two closing sections are high, meaning that the coverage across the sequence is fluctuating (see Table S3). Hence, it is not possible to draw a conclusion about a significant difference between the two versions. Nevertheless, when the strain possesses the pAsa8, the coverage of both junctions suggests their presence when we compare against the negative control. Since the SHY15-2939 strain does not have a pAsa8, the reads are not supposed to map on Junction 2. However, based on Table 2, we can detect a higher ratio than Junction 0, meaning that some reads map on the sequence. This is mainly due to the reads mapping on the tyrosine recombinase/integrase located on pAsa5, which was part of the sequence used for the junction analysis. Thus, the pAsa5 reads from the SHY15-2939 strain from this region could be detected during the analysis. Another interesting result is the high value of coverage for the pAsa5 gene against the chromosomal gene. The value of *P5G011* in all strains that bear pAsa8 is a little more than double the value of *dnaA*. For the *tetA* gene, the coverage is nearly identical to the *dnaA* gene, except for the M16474-11 strain. However, we cannot properly speculate any more about this analysis due to the high standard deviation (Table S3). The coverage analysis for the closure of pAsa5 does not adequately account for the lower signal observed in SHY18-3658 when compared to M16474-11 or SHY20-5455. There is no significant difference in coverage between the three strains that could justify the disparity in the PCR signal.

**Figure 4:**
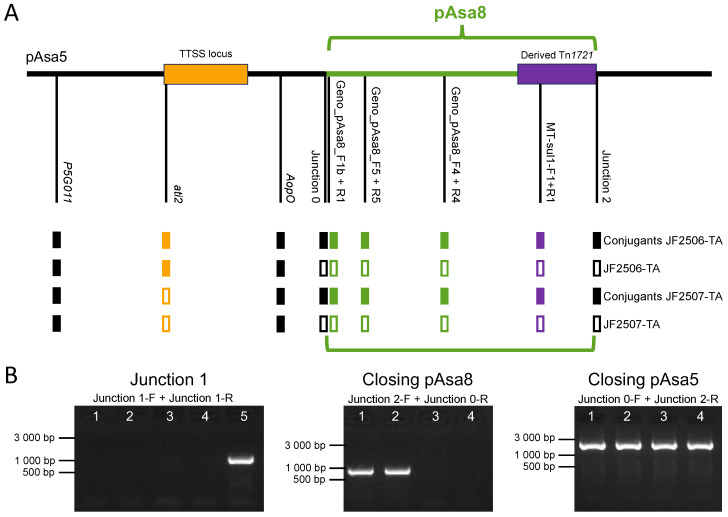
Genotyping of the transconjugants and the parental strains, JF2506-TA and JF2507-TA, after the conjugation assay. (**A**) Linear representation of the fusion between pAsa8 and pAsa5. The pAsa8 sequence is represented in green, while the pAsa5 sequence is in black. Below the sequence, PCR target locations are shown across the plasmid. Full rectangles indicate positive PCR signals for the corresponding DNA target, and white rectangles indicate negative results. The PCR results of each target are shown in Figure S2. (**B**) Junction 1 is absent in transconjugants, although plasmid self-closures of pAsa5 and pAsa8 are detectable. These PCR targets cannot be depicted in Figure panel A. The JF2506-TA and JF2507-TA transconjugants (lanes 1 and 2) compared to their parental strains, JF2506-TA and JF2507-TA (lanes 3 and 4). The fifth well for Junction 1 corresponds to the SHY16-3432 strain, which is a positive control.

To complete this study of the interaction between pAsa5 and pAsa8, we investigated whether it was possible to observe a fusion between the two plasmids in a strain of *A. salmonicida* subsp. *salmonicida* that had acquired pAsa8. This is conceivable, given the presence of functional genes for conjugation in pAsa8, as demonstrated by conclusive preliminary attempts to conjugate pAsa8 to a strain of *E. coli* (Massicotte, personal comm.). To achieve this conjugation between two strains of *A. salmonicida* subsp. *salmonicida*, the challenge was linked to the presence of a large number of ARGs in pAsa8, preventing us from having a selection marker for our transconjugant cells. We selected two strains from Europe (JF2506 and JF2507) with different genetic backgrounds to the strains in which pAsa8 has already been found (e.g., absence of the *AsaGEI1a* genomic island in these strains compared with the donor strain; Figure S2). From the JF2506 and JF2507 strains, we generated thermoadapted strains (JF2506-TA and JF2507-TA) that can grow at a higher temperature than the donor strain, thus providing a means of selecting transconjugants via growth temperature (see Material and Methods section). Another important point is that the recipient strains used were selected to have one strain with a complete pAsa5 (JF2506-TA) and another whose pAsa5 plasmid does not contain the TTSS (JF2507-TA) (Figure 4). We wanted to see if this had any impact on the interaction between pAsa5 and pAsa8.

The results of the conjugation showed that it was possible to transfer the pAsa8 plasmid into other strains of *A. salmonicida* subsp. *salmonicida* bearing pAsa5. Indeed, a PCR analysis showed that pAsa8 genes were present in both recipient strains (Figure 4). Once pAsa8 was acquired by the bacterium, the PCR results also showed a fusion between pAsa5 and pAsa8, as observed in other strains that naturally have pAsa8. In addition, as in the case of strains with pAsa8, a PCR analysis of transconjugants confirms the presence of fused and unfused plasmid forms in the bacterial population (Figure 4). We also tested if the conjugation of pAsa8 could result in the transposition of the derived Tn*1721* and the deletion of the pAsa8 genes, as shown in the SHY16-3432 strain. However, there was no PCR amplification for Junction 1 in any of the ten conjugants tested for both JF2506-TA and JF2507-TA.

## 4. Discussion

Plasmids are important vectors of horizontal gene transfer, often providing beneficial traits to the bacteria and enhancing host fitness [27]. This study demonstrated the presence of replicons corresponding to the fusion between the pAsa8 and pAsa5 plasmids. The PCR results and pAsa8 conjugation assays verified their ability to interact together within *A. salmonicida* subsp. *salmonicida* cells. Moreover, the pAsa5-3432 variant, characterized by the presence of the derived Tn*1721* transposon from pAsa8, appears to be an exceptional case that is not observed in other strains carrying pAsa8 or those obtained through conjugation, as demonstrated in this study.

The mechanisms and molecular partners responsible for the fusion of these two plasmids remain to be determined. However, we identified the presence of two tyrosine-type recombinases/integrases on pAsa5 and pAsa8 that share a high degree of similarity, with an 87% shared identity percentage in amino acid sequences. Both genes encoding these proteins are located in proximity to each junction (Junction 0 and Junction 2); in other words, they are close to the 22-nucleotide sequence used for the plasmid fusion. This suggests that these genes may play roles in the fusion process. One possible hypothesis is that the 22-nucleotide sequence could serve as a recognition site for these two recombinases and thus enable the fusion of the two plasmids.

We aimed to investigate the presence of the 22-nucleotide sequence at various locations within both plasmids and in the *A. salmonicida* subsp. *salmonicida* chromosome. In the case of pAsa5, the 22 nucleotides are exclusively found at one site within the plasmid, corresponding to the insertion site of pAsa8. However, in pAsa8, the majority of this sequence can be found at three different locations. As expected, the complete 22-nucleotide sequence is present in the pAsa8 site involved in recombination with pAsa5, according to the pAsa8 sequence from the M16474-11 strain. Additionally, 18 of the 22 nucleotides can be found at around 4 kbp, where Junction 1 is located in pAsa5-3432. Sixteen of the twenty-two nucleotides are found on pAsa8 at around 8 kbp from Junction 1. When we analyzed the chromosome of a reference genome of *A. salmonicida* subsp. *salmonicida* (GenBank NC_009348.1), no significant results were found, suggesting the absence of the 22-nucleotide sequence in the chromosome.

It cannot be ruled out that pAsa8 may interact with plasmids other than pAsa5. However, to date, no other *A. salmonicida* subsp. *salmonicida* plasmid containing the 22-nucleotide sequence was identified. On the other hand, the sequence has been found on an *A. hydrophila* plasmid named pAh2111-2-KPC. This plasmid appears to be a variant of pAsa8 with similar conjugative genes but lacking the transposon. Notably, a similar tyrosine recombinase is encoded on pAh2111-2-KPC, sharing a 94.49% identity with the one found on pAsa8. According to our analysis, pAsa5 and pAsa8 interact specifically at their tyrosine recombinase site, including the 22-nucleotide sequence. Consequently, the presence of this highly similar sequence in pAh2111-2-KPC suggests a potential interaction with the pAsa8 if they coexist within the same cell.

Outside the *Aeromonas* genus, the complete 22-nucleotide sequence was found on one plasmid from *Citrobacter freundii* and one from *Enterobacter cloacae*. When we compare pAsa8 against these two plasmids, there are no significant similarities that could indicate that they are related to pAsa8. Also, a search for pAsa8 tyrosine recombinase homologs failed to identify any significant results for *Citrobacter freundii* and *Enterobacter cloacae*. An interaction between those two plasmids and pAsa8, as it was observed in pAsa5, would be surprising since they lack the tyrosine recombinase. However, when we blast the plasmid of *C. freundii* and *E. cloacae* against the pAsa8, there are other identical regions that could be involved in homologous recombination events.

Plasmid fusion is a relatively rare and understudied event. Various genetic events involving mobile DNA elements, such as IS transposition, can be responsible for the fusion of two plasmids [28,29]. In some studies, the fusion can occur through homologous recombination [30,31]. In our case, the only sequence that is perfectly identical is the 22 nucleotide section, which is relatively short. However, in *E. coli*, the RecA protein can be involved in the recombination of short homologous sequences with a minimum of 20 nucleotides [32,33]. Since the recombination rate exponentially increases with the length of the sequence, we investigated whether there were other highly similar regions between pAsa8 and pAsa5. A BLASTN analysis between the two plasmids revealed a first region corresponding to the gene coding for the tyrosine recombinase/integrase mentioned earlier. This region has an identity percentage of 83% (871/1045 nucleotides) and is located right next to the recombination site in both plasmids. It cannot be ruled out that the similarity between the two recombinases could lead to homologous recombination. This genetic event could explain the complete insertion of plasmid pAsa8 at Junction 2.

To explore this hypothesis further, we examined whether the pAsa5-3432 variant could also be explained by homologous recombination. This variant contains only the Tn*1721* transposon and a region of 5 kbp that has been deleted. The first homologous recombination event may have occurred at Junction 2, involving the similar tyrosine recombinase/integrase found in pAsa8. This recombination would result in the insertion of the entire pAsa8 at Junction 2 of the Tn*1721*. Subsequently, we looked for another region at the end of the transposon that could be the target of a second homologous recombination event that would result in the loss of the pAsa8 backbone. A further BLASTN analysis between pAsa8 and pAsa5-3432 revealed a similar region of potential interest for the recombination at Junction 1. The region on pAsa5-3432 is located exactly at Junction 1 and has an identity percentage of 87.6%. While homologous recombination could therefore explain the 5 kbp deletion on pAsa5, this region on pAsa8 is positioned 4 kbp further from Junction 1, meaning that homologous recombination with this region should have left this 4 kbp sequence on pAsa5-3432, which is not the case. Therefore, the pAsa5-3432 variant does not appear to be explained by a double homologous recombination event, but more likely by an aberrant second recombination event following the initial fusion of both plasmids at Junction 2.

Another interesting result from this study is the coexistence of both of the independent versions of pAsa5 and pAsa8, alongside the fused version in *A. salmonicida* subsp. *salmonicida*. To determine the prevalence of these forms, we conducted a sequencing reads analysis. The results revealed significant variability in gene coverage, indicating a heterogeneous population. Indeed, the value of the standard deviations for junctional coverage is too large to be able to draw any significant conclusions about the predominant form. When the chromosome coverage is compared, the *P5G011* gene on pAsa5 appears to be present in two copies. This result is surprising given that the gene’s coverage is only present in one copy for control strains (without pAsa8). Moreover, in previous studies, it was already demonstrated that pAsa5 is present in one copy in the cell [10]. While speculative, this suggests that strains carrying pAsa8 may have an increased number of pAsa5 copies per cell, potentially leading to a higher quantity of TTSS, which could enhance virulence.

It is important to note that the fused versions of pAsa8 and pAsa5 may be different from the addition of both complete versions. Since both pAsa5 and pAsa8 genes are present, we assume that this is a fusion between the two whole plasmids. However, it cannot be ruled out that different versions of pAsa5 or pAsa8 could be fused, as demonstrated with the conjugation of the JF2507-TA strain (where pAsa5 does not have the TTSS segment).

Conjugation assays demonstrate that the fusion of pAsa8 and pAsa5 can occur within other strains of *Aeromonas salmonicida* subsp. *salmonicida* than the strains coming from Quebec that naturally carry pAsa8. With the presence of Junction 0 and Junction 2 for the JF2506-TA and JF2507-TA strains, we can demonstrate that pAsa5 fuses with pAsa8 after the latter’s insertion.

In this study, we provided new data, further demonstrating the complexity of pAsa5 biology. In the past, the large number of insertion sequences present on this plasmid and their roles in different recombination scenarios for plasmids (loss of TTSS and fusion with pAsa9) were highlighted [13]. This time, we demonstrate that pAsa5 has similar sequences to pAsa8, and this is the cause of the fusion between the two plasmids. We also demonstrated the conjugative capacities of the pAsa8 plasmid. Considering the numerous antibiotic resistance genes it carries, the conjugative ability of pAsa8 poses a risk for ARG dissemination. It would not be surprising to find this plasmid or variants of it in other bacterial species. The pAsa8-b plasmids found in the *Aeromonas caviae* strains, R25-2 and pAh2111-2-KPC, and in the *Aeromonas hydrophila* strain, Ah2111, are variants of pAsa8, but neither one bore the transposon carrying the ARGs. Investigating the evolutionary relationships among these plasmids will provide further insights into this plasmid family. The discovery of new variants of pAsa8 in the future will contribute to a better understanding of this plasmid group.

## Figures and Tables

**Table 1:** *A. salmonicida* subsp. *salmonicida* strains used in this study.

Strain Name	Origin	Commentary	References
M15448-11 ^a^	Canada	Bears pAsa8.	[9]
M16474-11 ^a,b^	Canada	Bears pAsa8.	[9]
SHY16-3432 ^b^	Canada	Bears pAsa5-3432.	[8]
SHY18-3658 ^a,b^	Canada	Bears pAsa8.	This study
SHY20-5455 ^a,b^	Canada	Bears pAsa8.	This study
SHY21-2359 ^a^	Canada	Bears pAsa8.	This study
SHY21-3158 ^a^	Canada	Bears pAsa8.	This study
JF2506-TA	Norway (parent strain)	Recipient strain with pAsa5 that bears TTSS. This strain is derived from JF2506. References are made to the parent strain.	[16,17]
JF2507-TA	United Kingdom (parent strain)	Recipient strain with pAsa5 that does not bear TTSS. This strain is derived from JF2507. References are made to the parent strain.	[16,17]
A449 ^b^	France	Negative control for PCR experiments. Does not bear pAsa8.	[14]

^a^ These strains also carry the classic plasmidome (pAsa1, pAsa2, pAsa3, pAsal1, and pAsa5). ^b^ The strain for which the genome sequence is available.

**Table 2:** Relative read abundance for different strains of *A. salmonicida* subsp. *salmonicida*. In this table, the read coverage values of the different genetic elements analyzed (genes and junctions) were normalized by considering the chromosomal gene (*dnaA*) at a value of 1.0 to simplify the comparison. The means and standard deviations of the raw coverage values are shown in the Supplementary Data (Table S3).

Name of the Gene	Length (pb)	Ratio for the Strains			
		M16474-11	SHY18-3658	SHY20-5455	SHY15-2939
*dnaA*	1371	1.00	1.00	1.00	1.00
*P5G011*	534	2.16	2.45	2.27	0.98
*tetA*	1200	2.36	1.05	1.25	0.00
Closing of pAsa5	801	1.79	1.40	1.28	0.57
Closing of pAsa8	801	1.82	0.90	0.90	0.05
Junction 0	757	0.85	0.34	0.59	0.05
Junction 2	804	1.44	0.70	0.56	0.28

## Data Availability

The data presented in this study are available in the article’s figures and tables and in the Supplementary Materials. The raw data presented in this study are available on request from the corresponding author.

## Supplementary Materials

The following supporting information can be downloaded at https://www.mdpi.com/article/10.3390/microorganisms11112685/s1, Figure S1: The growth protocol used to develop thermoadapted strains of *A. salmonicida* subsp. *salmonicida*; Figure S2: PCR confirmation for the conjugation assay of pAsa8; Table S1: Primer used in this study; Table S2: PCR conditions used in this study; Table S3: Raw analysis of read coverage for different strains of *A. salmonicida* subsp. *salmonicida*.
