# Supplementary material for: Interaction of pAsa5 and pAsa8 Plasmids in Aeromonas salmonicida subsp. salmonicida
Source: Microorganisms. 2023 Nov 2;11(11):2685. doi: 10.3390/microorganisms11112685 (PMC10673383; doi:10.3390/microorganisms11112685)
Supplement: Supplementary file 1 [file microorganisms-11-02685-s001.zip › VF_Supplementary_material.pdf]

Supplementary material

Interaction of pAsa5 and pAsa8 plasmids in *Aeromonas salmonicida* subsp. *salmonicida*

Pierre-Étienne Marcoux[1,2*], Sarah B. Girard[1,2], Kim C. Fournier[1,2], Catherine A. Tardif[1,2], Ariane Gosselin[1,2], Steve J. Charette[1,2,3*]

1. Institut de Biologie Intégrative et des Systèmes (IBIS), Université Laval, Quebec City, QC, Canada, G1V 0A6

2. Département de biochimie, de microbiologie et de bio-informatique, Faculté des sciences et de génie, Université Laval, Quebec City, QC, Canada, G1V 0A6

3. Centre de recherche de l'Institut universitaire de cardiologie et de pneumologie de Québec (IUCPQ), Quebec City, QC, Canada, G1V 4G5

[*]To whom correspondence should be addressed: Institut de Biologie Intégrative et des Systèmes (IBIS), Pavillon Charles-Eugène-Marchand, 1030 avenue de la Médecine, Université Laval, Quebec City, QC, Canada, G1V 0A6. ORCID : 0000-0002-0199-2852.

pierre-etienne.marcoux.1@ulaval.ca and steve.charette@bcm.ulaval.ca; Telephone: 1-418-656-2131, ext. 406914

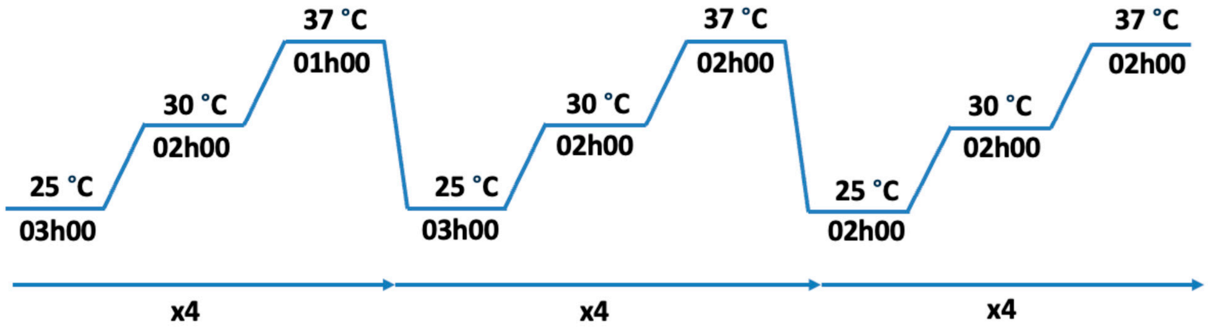

Figure S1. The growth protocol used to develop thermoadapted strains of *A. salmonicida* subsp. *salmonicida.* Both parental strains, JF2506 and JF2507, undergo 12 cycles of incubation in tryptic soy broth at various temperatures and time intervals. At the end of this protocol, the strains can grow at higher temperatures (30 °C to 35 °C) than their optimal condition, which is around 18 °C.

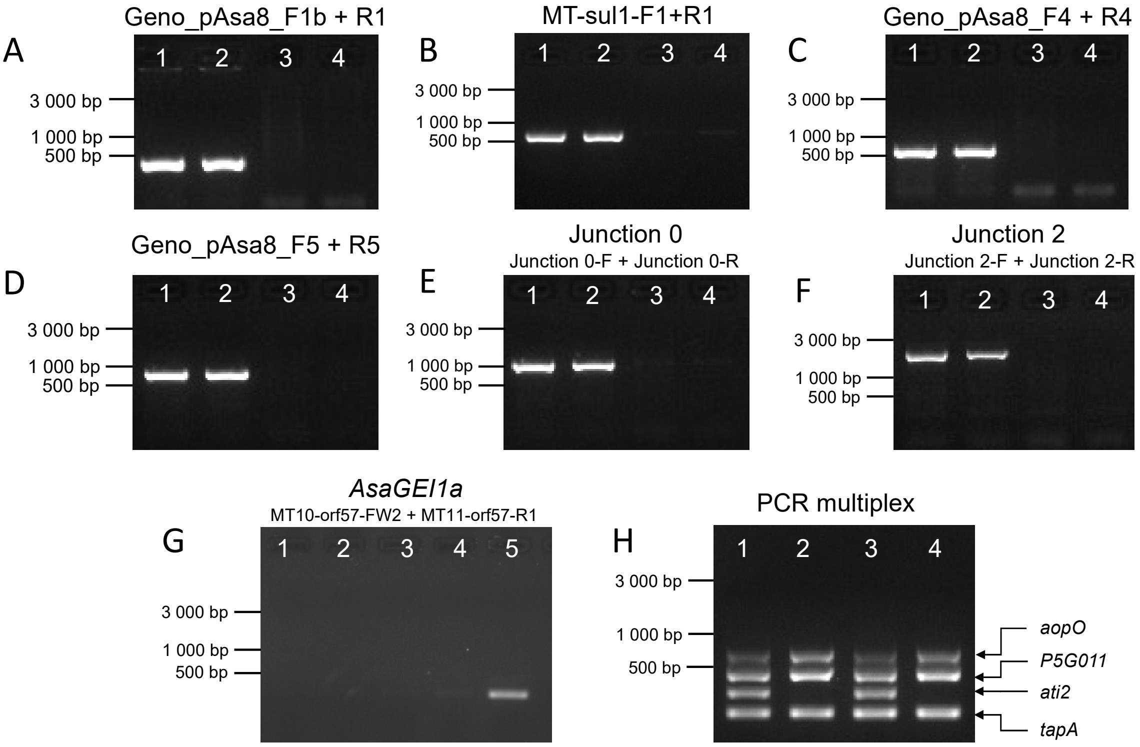

Figure S2. PCR confirmation for the conjugation assay of pAsa8. This figure presents an example of the PCR analyzes which were carried out on the conjugants obtained from the parental strains JF2506-TA and JF2507-TA to confirm the presence of the pAsa8 plasmid (A to D), its fusion with the pAsa5 plasmid (E and F) as well as the presence of *AsaGEI1a* (G) and the pAsa5 plasmid in its complete form or without TTSS (H). In each image, the lanes 1 and 2 correspond to the transconjugants JF2506-TA and JF2507-TA, while lanes 3 and 4 represent the parental strains JF2506-TA and JF2507-TA. The fifth well corresponds to the positive control which is the donor strain SHY20-5455.

Table S1. Primers used in this study.

Primer	Sequence (5' -> 3')	Reference
**PCR to verify the insertion of pAsa8 into pAsa5**		
Junction 0-F	GTGTGGTCAATCCCTGTGTATAG	This study
Junction 0-R	CACTTCATGGGCCAAGAGATAA	This study
Junction 2-F	CCATGGCGATCAGTTCAGGGATAAGG	[1]
Junction 2-R	GAATGATGCTGGCGGTACTGACTGAG	[1]
Junction 1-F	CTTGGCTATCAGGGATGCGAACTTG	[1]
Junction 1-R	CAGCCAGCGATTGATGGTCTTGAAC	[1]
**PCR to verify the absence of lost genes just like in pAsa5-3432**		
KrfA-F	CTCATAGTCAGCTTCGATCCGATCG	[1]
KrfA-R	GTTGGAAACGGCACAAGAGCAGA TC	
HP1-F	CAGTTATCGGACAATTCGGTACGG	
HP1-R	CAATCGTAGAACTTGCCTCCTTCC	
HP2-F	CTTGTTTGCCCTTTGGATCGGATGAC	
HP2-R	GGCCAACATGAATGCGATGAAGTACC	
**PCR to detect pAsa8**		
Geno_pAsa8_F1b	CATGATGGTCACACCTCGATACTC	[2]
Geno_pAsa8_R1	GCAATCCACTACTCAGTAGGTGAG	
MT-sul1-F1	GGGCTACCTGAACGATATCC	
MT-sul1-R1	CTAGGCATGATCTAACCCTCG	
Geno_pAsa8_F4	GTGATGATGTGAAATCTGCGTGGG	
Geno_pAsa8_R4	GCACTATCGCTTGCTTTCACCAGG	
Geno_pAsa8_F5	GCTTGCGGTCTACAGTGATATCTC	
Geno_pAsa8_R5	CGGTACTCAAATGCAACGCTACTAC	
**Multiplex PCR to verify pAsa5 genes and a chromosomal control gene**		
aopO-1836F	GGTAACGTGGTCTTTGATAAGC	[3]
aopO-2507R	GTTGAAACTCTTGATCTGACCC	
P5G011-86F	ACAATTACATCATTCTGGTTGGC	
P5G011-546R	AGAAATCTCGTCAACATCAATCG	
ati2-732F	CACTTCAATGTCTGTTTTTGCG	
ati2-1052R	TACATAACGTCAGAACCTTCAGC	
tapA-217F	GTTAGTTCCTGCATCACATCC	
tapA-406R	CCTTATTGAACTGATGATCGTGG	
**PCR to detect *AsaGEI***		
MT10-orf57-FW2	AACCATCATCGAGGCGGAGTTCTT	This study
MT11-orf57-R1	ATCCGGTGAGTTGGTGTCCATGAT	[4]

Table S2. PCR conditions used in this study.

Target	Primers used	Initial denaturation	30 Cycles			Final extension
			Denaturation	Annealing	Extension	
Junction 0	Junction 0-F + Junction 0-R	2 m 30 s at 95°C	30 s at 95°C	30 s at 55 °C	1 m at 68 °C	5 m at 68 °C
Junction 1	Junction 1-F + Junction 1-R	2 m 30 s at 95°C	30 s at 95°C	30 s at 55 °C	1 m at 68 °C	5 m at 68 °C
Junction 2	Junction 2-F + Junction 2-R	2 m 30 s at 95°C	30 s at 95°C	30 s at 60 °C	1 m at 68 °C	5 m at 68 °C
Closing of pAsa5	Junction 0-F + Junction 2-R	2 m 30 s at 95°C	30 s at 95°C	30 s at 58 °C	1 m 45 s at 68 °C	5 m at 68 °C
Closing of pAsa8	Junction 2-F + Junction 0-R	2 m 30 s at 95°C	30 s at 95°C	30 s at 55 °C	1 m at 68 °C	5 m at 68 °C
pAsa8 genes	Geno_pAsa8	2 m 30 s at 95°C	30 s at 95°C	30 s at 55 °C	30 s at 68 °C	5 m at 68 °C
pAsa5 genes	PCR multiplex	2 m 30 s at 95°C	30 s at 95°C	30 s at 55 °C	1 m at 68 °C	5 m at 68 °C
*AsaGEI*1*a*	MT10-orf57-FW2 + MT11-orf57-R1	2 m 30 s at 95°C	30 s at 95°C	30 s at 60 °C	30 s at 68 °C	5 m at 68 °C

Table S3. Raw analysis of read coverage for different strains of *A. salmonicida* subsp. *salmonicida*.

Name of the genes	Length (pb)	M16474-11		SHY18-3658		SHY20-5455		SHY15-2939	
		Mean	Standard deviation	Mean	Standard deviation	Mean	Standard deviation	Mean	Standard deviation
dnaA	1 371	66.79	10.94	105.97	6.43	103.01	13.41	172.71	18.65
P5G011	534	144.16	11.79	259.87	13.11	234.27	39.07	169.48	12.42
tetA	1 200	157.72	16.05	111.09	12.12	128.88	26.49	0	0
Closing of pAsa5	801	119.67	64.86	148.65	98.85	131.84	115.45	99.15	43.44
Closing of pAsa8	801	121.83	90.37	95.48	77.31	92.83	65.72	7.81	14.64
Junction 0	757	56.94	37.29	35.96	24.27	60.36	37.15	9.12	9.19
Junction 2	804	96.17	64.17	77.24	61.64	57.57	46.55	47.63	35.05

REFERENCES

1. Massicotte, M.A.; Vincent, A.T.; Schneider, A.; Paquet, V.E.; Frenette, M.; Charette, S.J. One *Aeromonas salmonicida* subsp. *salmonicida* isolate with a pAsa5 variant bearing antibiotic resistance and a pRAS3 variant making a link with a swine pathogen. *Science of the Total Environment* **2019**, *690*, 313-320, doi:10.1016/j.scitotenv.2019.06.456.

2. Trudel, M.V.; Vincent, A.T.; Attere, S.A.; Labbe, M.; Derome, N.; Culley, A.I.; Charette, S.J. Diversity of antibiotic-resistance genes in Canadian isolates of *Aeromonas salmonicida* subsp. *salmonicida*: dominance of pSN254b and discovery of pAsa8. *Scientific Repports* **2016**, *6*, 35617, doi:10.1038/srep35617.

3. Marcoux, P.E.; Vincent, A.T.; Massicotte, M.A.; Paquet, V.E.; Doucet, E.J.; Hosseini, N.; Trudel, M.V.; Byatt, G.; Laurent, M.; Frenette, M.; et al. Systematic analysis of the stress-induced genomic instability of type three secretion system in *Aeromonas salmonicida* subsp. *salmonicida*. *Microorganisms* **2020**, *9*, 1-10, doi:10.3390/microorganisms9010085.

4. Emond-Rheault, J.G.; Vincent, A.T.; Trudel, M.V.; Brochu, F.; Boyle, B.; Tanaka, K.H.; Attere, S.A.; Jubinville, E.; Loch, T.P.; Winters, A.D.; et al. Variants of a genomic island in *Aeromonas salmonicida* subsp. *salmonicida* link isolates with their geographical origins. *Veterinary Microbiology* **2015**, *175*, 68-76, doi:10.1016/j.vetmic.2014.11.014.
